# Cardiorespiratory Fitness Is Associated With Drop Out From Sport in Norwegian Adolescents. A Longitudinal Study

**DOI:** 10.3389/fpubh.2020.502307

**Published:** 2020-12-04

**Authors:** Ivar Fossland Moa, Sveinung Berntsen, Pål Lagestad

**Affiliations:** ^1^Faculty of Education and Arts, Nord University, Levanger, Norway; ^2^Department of Sport Science and Physical Education, University of Agder, Kristiansand, Norway

**Keywords:** adolescence, cardiorespiratory fitness, organized sport, drop out, longitudinal

## Abstract

Several studies indicate that participation in organized sport may result in higher physical activity levels among youth which are associated with high levels of cardiorespiratory fitness. However, no study has examined whether cardiorespiratory fitness (VO_2peak_) is associated with drop out from sport. The study was a 5-year longitudinal study which followed a sample of adolescents, with measures of cardiorespiratory fitness, weight and height between the age of 14 and 19 yrs. Self-reported data about participation in sport, active commuting and physical activity level were also included. Through logistic regression analyses we found a positive association between cardiorespiratory fitness at the age of 14 years and participation in organized sport at the age of 19. However, no significant associations were found between physical activity (PA) level, overweight, gender and active commuting to school at the age of 14, and participation in organized sport at the age of 19. We argue that a high level of cardiorespiratory fitness may increase the probability for experiencing high levels of enjoyment, competence and performance in sport, because sport participation requires a certain level of cardiorespiratory fitness. The findings indicates the importance of removing barriers, and to increase access and design of sport programs of interest to youth in the contexts in which they live, attract adolescents with different levels of ambition and abilities in sport. Further studies should include longitudinal studies among young children, and their drop out patterns from sport during adolescence.

## Introduction

Several studies indicate that participation in organized sport seems especially important in relation to high physical activity (PA) levels which are associated with high levels of cardiorespiratory fitness (1–6). A longitudinal study among adolescents found that adolescents participating in organized sport, had significantly higher VO_2_peak values than adolescents participating in unorganized PA, and those with <1-h weekly PA - at both 14 and 19 years of age (7). Joining organized youth sports at an early age, and continuing through adolescence, appears to increase the likelihood of a physically active lifestyle in young adulthood (8). A 21-year longitudinal study showed that persistent participation in youth sport was associated with adult physical activity level (8), later confirmed by other studies (9–11). These findings point toward the importance of maintaining a physical activity level during childhood and adolescence. Regular participation in PA during adolescence has numerous health benefits, including reduced probability for obesity, bone health, psychological health, and cardiorespiratory fitness (12–15).

In Norway, more than 80% of children participate in organized sport, and most start before 10 years of age (16). However, there is a high drop out rate from organized sport with increasing age in Norway as in other countries (16–18). Several researchers have explored potential factors that may be associated with drop out from sport, and there seem to be many potential explanations for drop out. In a systematic review focusing on drop out from organized sport, five major factors were associated with dropout; lack of enjoyment, perceptions of competence, social pressures, competing priorities and physical factors such as maturation and injuries (19). In another review results showed that the players dropping out from soccer, felt that time demands, especially time used in traveling to compete, were onerous (18). Other factors associated with drop out were lower perceptions of competence and lack of fulfillment of basic psychological needs.

Contextual factors associated with drop out were poor relationships with teammates or coaches, lack of opportunity to play, competing time demands, and later birthdate in relation to competitive year (18). This particular study also pointed to lack of enjoyment as a drop out factor. Correspondingly, research indicated that adolescents' enjoyment of organized sports training decreased from the age of 14 to the age of 19 (20). In a study of ice hockey players, it was found that players from larger cities were more likely to drop out (21). Drop out has also been associated with physical limitations of body size and skeletal development (22). In Patriksson's study (23), the main reasons for drop out were that the adolescents did not like the coach, they found other sports more fun, and feeling of bad (low) performance in the sports activity. Other studies found that, compared with those who continued with their sports, dropouts were less task-oriented (24).

Research has suggested that among families with lower family socioeconomic status, the father's role may be important to promote youth to sustain sports participation (25). Other studies found that low perceived competence and low performance were associated with increased drop-out (23, 26). However, even if there are several earlier studies examining factors associated with drop out from sport, no study has examined whether VO_2peak_, PA level, overweight, gender and active commuting to school are associated with drop out from sport, using a longitudinal design. A study indicated that these are factors that could be associated with drop out from sport (27). The importance of cardiorespiratory fitness in relation to dropping out from sport could be ascribed to the strong influence of physical factors in relation to performance in some sports, in which biological-maturity and status of height, weight, strength, and power constitute a major element in performance capacity. The present study aimed to determine if VO_2peak_, as well as PA level, overweight, gender and active commuting to school are associated with drop out from sport among Norwegian adolescents.

## Materials and Methods

### Design

This 5-year longitudinal study followed a sample of adolescents from the age of 14 to 19 years, with yearly measures of cardiorespiratory fitness, weight and height as well as self-reported participation in sport, active commuting and physical activity level. Peak oxygen uptake, weight and height were measured in the period February to April each year where the students were at the end of 8th grade (14 years old), 9th grade (15 years old), 10th grade (16 years old), and also in the 1st year (17 years old), 2nd year (18 years old), and third year (19 years old) at high school.

### Subjects

Six 8th grade school classes (two classes in each of three groups) from upper secondary schools in a medium sized town in the middle of Norway with 144 students, were randomly selected for the present study. Of these, 124 students accepted to participate in the study, but only 48 participants (28 boys and 19 girls) completed both the pretest (in 2010) and the retest (in 2015), and had valid data on all measurements. The subjects were informed verbally about the aim of the study, as well as methodology before inclusion in the present study. A written consent was signed each year by the parents (14 and 15 years of age) and by the students (16–19 years of age) according to the ethical regulations of research (NSD and the Norwegian ethical regional committee (id: 488715, 2014).

### Procedures

All the tests were carried out by the same test leader, in the same laboratory, with the same equipment, and with the same procedures, and at the same time of the day (during school hours). The test were also carried out in the same period (April and May) each year. Peak oxygen uptake was determined while running on a treadmill (Woodway S5, Woodway Inc., Waukesha, USA) until exhaustion. In advance, the students had been informed about the procedures and the test conditions (abstain from vigorous intensity physical activity the day before test, food intake 2–3 h before test, only participate in light physical activity in physical education class if they had physical education on the same day before the test). Subjects were tested in training pants or shorts, t-shirt and running shoes. Oxygen uptake was measured by OxyCon Pro (Erich Jaeger GmbH, Hoechberg, Germany), and using a test protocol with an inclination set to 10.5%. Before the test, the students were asked about their physical activity patterns. Inactive girls, or girls with overweight, started the test with 4 km/h, girls who exercised 1–2 days a week started the test with 4 km/h, while girls who exercised 3–4 days a week started the test with 5 km/h. Boys used the same procedures, but with 1 km/h higher initial speed. The speed increased with 1 km/h every minute. Peak oxygen uptake was defined as when the oxygen uptake achieved a flattening/decrease of the oxygen uptake curve with increased load (the respiratory exchange ratio > 1.00). Mean oxygen uptake of the two highest measures after 5 and 6 min was registered as peak oxygen uptake. The test lasted for 5–6 min.

Height was measured with a stadiometer (kawe medizintechnik seit 1890), permanently attached to the wall. The subjects did not wear shoes, and height was converted to the nearest centimeter. Weight was measured with a Seca Digital weight (gmbh & co., Germany, model 877, accuracy of 0.1 kilo). Body mass index was calculated by international standards. Overweight was calculated in relation to international standards, where the cut-off values for overweight were set at 22.62 for boys and 23.34 for girls at 14 years of age, 23.90 for boys and 24.37 for girls at 16 years of age, and 25 for all adolescents at 19 years of age (28).

The following question about physical activity (29) was included: “How many days a week are you so physically active that you become sweaty or out of breath?” The response options were “never,” “1 day a week,” “2–3 days a week,” “4–5 days a week,” and “6–7 days a week.” This variable was dichotomized into a variable with “ <6–7 days a week,” and “6–7 days a week.” In addition, the adolescents answered a question about active commuting to school, with the response options “yes” or “no.” Sports participation was examined using the question: “During the season, how often do you participate in organized sport?” The response options were: “Never, rarely,” “1–3 days a month,” “1 day a week,” “2–3 days a week,” “4–6 days a week,” “every day.” The cut-off for sports participation was set at weekly participation.

### Statistical Analysis

Descriptive characteristics are presented as mean and standard deviation (SD). Logistic regression was performed to calculate Odds Ratios (OR) with 95% Confidence Intervals (CI) for sports participation at 19 years of age as the outcome variable and gender, VO_2peak_, active commuting to school, participation in PA and overweight as independent variables. Due to multi-colinearity, sports participation at 14 years of age was not included in the model. Variables associated with participation were included in the multivariate logistic regression analyses if *p* < 0.2. Dependent variables were then removed in a step-down fashion, until statistical significance (*p* ≤ 0.05) was reached for the remaining variables (30). Statistical analysis was performed with SPSS statistical software version 24 (SPSS Inc., Chicago, IL, USA).

## Results

The aim of the study was to determine if VO_2peak_, as well as PA level, overweight, gender and active commuting to school are associated with drop out from sport among Norwegian adolescents.

Table 1 shows baseline characteristics of the study population at baseline (14 years of age) according to; age, height, weight, body mass index (BMI), percentage with overweight, and VO_2peak._.

**Table 1:** Baseline characteristics of the subjects who completed the retest at the end of high school (19 years of age): Age, height, weight, (kg), body mass index (BMI), percentage with overweight (%), and VO_2peak_ (mL/kg/min).

	**Boys (*****n*** **=** **28)**		**Girls (*****n*** **=** **19)**	
	**Mean**	**SD**	**Mean**	**SD**
Age^a^ (yrs)	14	0.5	14	0.5
Height (m)	166.2	10.3	161.2	7.2
Weight (kg)	53.9	10.2	55.5	14.5
BMI (kg/m^2^)	19.4	3	21.1	4.5
Overweight, %	14.3		21.1	
VO_2peak_ (mL/kg/min)	53.8	6.1	45.8	8

a *Age is calculated from year at school*.

Table 2 shows baseline characteristics of the study population at 14 years of age according to; reported activity level, active commuting to school, participation in organized sport and gender.

**Table 2:** Baseline characteristics of independent variables included in the analyses of the study among the subjects who completed the retest at the end of high school (19 years of age).

	**Boys**	**Girls**
**N**	**28**	**19**
Reported physical activity level		
4 days a week or more, %	57.1	31.6
<4 days a week, %	42.9	68.4
Active commuting to school		
Yes, %	53.1	47.6
No, %	46.9	52.4
Participation in organized sport		
Weekly, %	82.1	68.4
Less than weekly, %	17.9	31.6
Gender, %	59.6	41.4

Table 3 shows included variables (measured at the age of 14) and their association with participation in sport at the age of 19. The initial analyses show that both VO_2peak_, gender and self-reported physical activity level at the age of 14 were significantly associated with participation in organized sport at the age of 19. However, in the multivariate analyses, only VO_2peak_ was significantly associated with participation in organized sport at the age of 19. The results show that adolescents who still participate in organized sport at the age of 19, have 13% higher VO_2peak_ than adolescents who drop out from organized sport.

**Table 3:** Factors associated with participation in sports at 19 years of age.

	**Bi-variate**		**Multi-variate**	
	**OR (95%CI)**	***P*-values**	**OR (95%CI)**	***P*-values**
VO_2peak_ (mL/kg/min)	1.13 (1.06, 1.21)	<0.001	1.13 (1.06, 1.21)	<0.001
Girls	0.32 (0.13, 0.78)	0.012	–	
Active commuting to school	1.05 (0.42, 2.62)	0.921	–	
Physically active, 6–7 days a week	2.57 (1.01, 6.54)	0.047	–	
Overweight	0.36 (0.09, 1.43)	0.148	–	

**Figure 1:**
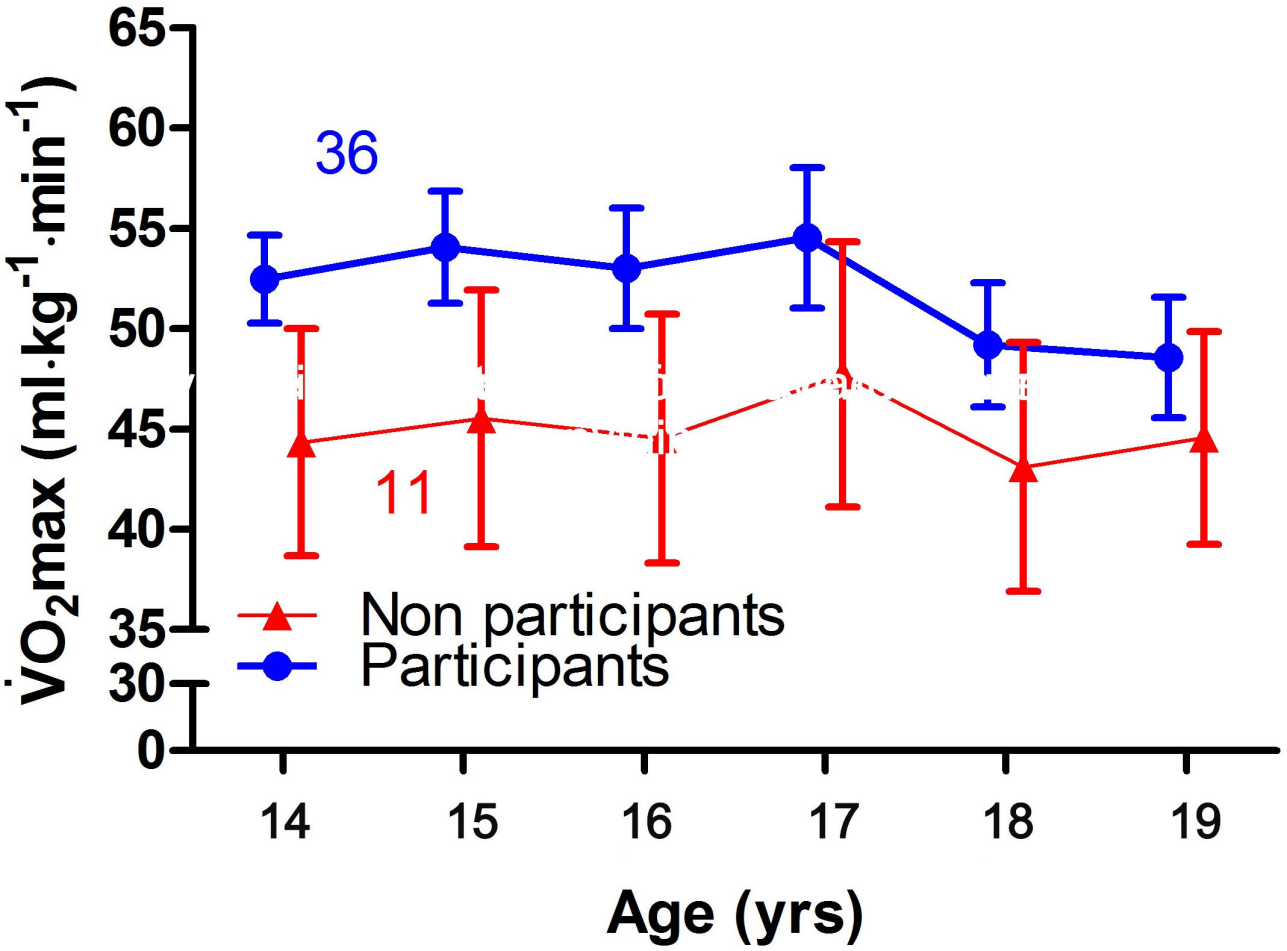
Mean and 95% CI of VO_2peak_ (mL/kg/min) among participants and non-participants in organized sport among the subjects that completed the re-test at the end of high school, from the age of 14 to the age of 19.

Figure 1 shows the development of VO_2peak_ among participants and non-participants in organized sport among the subjects who completed the retest at the end of high school, from the age of 14 to the age of 19. Figure 1 shows that the VO_2peak_ was higher among participants in organized sport than non-participants in organized sport at the age of 14.

## Discussion

The main finding of the present study was that cardiorespiratory fitness at the age of 14 was associated with participation in sport at 19 years of age. Neither overweight, self-reported physical activity level nor active commuting at 14 years of age were associated with participating in sport at 19 years of age.

Even though no study has examined the importance of cardiorespiratory fitness according to drop out from a longitudinal perspective, previous cross-sectional research has shown positive associations between cardiorespiratory fitness and participation in sport (1–5, 11, 27). Knowing that regular participation in organized sport through adolescence seems to increase the likelihood for a physically active lifestyle as adults (8, 10), it is somehow problematic that low levels of cardiorespiratory fitness are associated with drop out from sport. However, previous research about drop out from sport has identified other factors that can contribute to the understanding why high VO_2peak_ prevents drop out from sport. Studies have shown that lack of enjoyment was a major factor associated with dropout (18, 19). It may be argued that certain types of sports require a certain level of cardiorespiratory fitness (VO_2peak_), and that high levels of VO_2peak_ increase enjoyment and well-being during sport and prevent drop out. Less enjoyment among dropouts in organized sports training has also been found in a study (20). Studies also show that lower perception of competence (18, 19, 26) and bad performance (22, 23) were important factors associated with drop out from sport.

It is also appropriate to argue that a high level of cardiorespiratory fitness increases the probability of experiencing high levels of competence and performance in sport, because sport participation requires a certain level of cardiorespiratory fitness (VO_2peak_). Some studies support this argument (20, 27). It is reported that high levels of cardiorespiratory fitness increased performance and involvement in soccer (31), and that junior soccer players may benefit from aerobic training to attenuate the decline in short-passing ability in soccer (32).

The findings highlight the importance of organizing sport with exercises that increase cardiorespiratory fitness among youth, and that adolescents with high levels of VO_2peak_ have the tendency to continue to participate in organized sport It is also reported that young athletes have higher cardiorespiratory fitness than their untrained peers (33), but that young people can increase their cardiorespiratory fitness through exercise training (33–36). The research points to physical activity of a certain kind (endurance), duration, frequency and intensity as necessary to improve cardiorespiratory fitness with training (37). A study found that even a training period consisting of two periods of circuit training a week for 6 weeks, increased physical fitness among a group of adolescents (38). It is suggested that that efforts to promote moderate to vigorous PA among youths may provide long-term benefits, by helping to develop favorable exercise attitudes (5).

Another interpretation of the findings, is that there is a need to organize sport in a way that does not depend on high levels of cardiorespiratory fitness in order to perform and feel good, as highlighted in a study (27). This is in line with research pointing out that it is conceivable that sports clubs should offer activities that attract people with different levels of ambition and abilities, and should be organized to give all young people opportunities to develop (23). Given the apparent benefits of active participation, a study highlighted that it is important to remove barriers and increase access and, equally important, to design programs of interest to youth in the contexts in which they live (39).

### Strength and Limitations of the Study

The strengths of the study is that it is based on a longitudinal design among the same participants, at the same time of year, using the same questions and test every year, performed in the same test-lab/room, with the same test procedures, the same test equipment, and with the same test leader at all of the six test measures. Furthermore, many of the variables such as VO2_peak_, overweight and gender are based on high quality standard procedures. However, there are limitations in the study. The sample size is limited with a low number of participants at pre-test, and there was a large drop out at the post-test, causing a low response rate (38%). The homogeneity of the distribution is not known. Furthermore, the activity level is measured using self -reported data instead of objectively measures, which would have been preferable. Another criticism of the study is that it only includes six possible predictors of drop out from sport, and that other psychological variables may be better predictors of participation in sport.

## Conclusion

In the present study, we found an association between cardiorespiratory fitness (VO_2peak_) at the age of 14 and participation in organized sport at the age of 19. We did not find any significant association between PA level, overweight, gender and active commuting to school at the age of 14 years and participation in organized sport at the age of 19. We argue that a high level of cardiorespiratory fitness increases the probability of experiencing high levels of enjoyment, competence and performance in sport, because sport participation requires a certain level of cardiorespiratory fitness. To remove barriers, increase access and design sport programs of interest to youth in the contexts in which they live, seems important to attract people with different levels of ambition and ability in sport. Further studies should include more possible predictors of drop out from sport among adolescents and should also include more adolescents.

## Data Availability Statement

The datasets generated for this study are available on request to the corresponding author.

## Ethics Statement

The studies involving human participants were reviewed and approved by Norwegian ethical regional committee (id: 488715, 2014). Written informed consent to participate in this study was provided by the participants' legal guardian/next of kin.

## Author Contributions

IM has contributed to writing the introduction, discussion, conclusion, and also, a critical review of all the text during several drafts of the article and rewriting of the text. SB has contributed on design and methods and a critical review of the text during several drafts of the article. PL has contributed on design and methods, writing the introduction, methods, discussion, the conclusion, and a critical review of all the text during several drafts of the article and rewriting of the text. All authors contributed to the article and approved the submitted version.

## Conflict of Interest

The authors declare that the research was conducted in the absence of any commercial or financial relationships that could be construed as a potential conflict of interest.
